# Commentary on “Results of arthroscopic suspension fixation for acute posterior cruciate ligament avulsion fractures in adolescents: a retrospective cohort study”

**DOI:** 10.1097/JS9.0000000000003153

**Published:** 2025-08-05

**Authors:** Boyan Liu

**Affiliations:** a School of Pharmacy, China Medical University, Shenyang, China; b China Medical University and Queen University of Belfast joint College, China Medical University, Shenyang, China

*Dear Editor,*

I read with great interest the recent study by Chen *et al*, who reported promising outcomes using an arthroscopic suspensory fixation technique for acute posterior cruciate ligament (PCL) avulsion fractures in skeletally immature patients[1]. Their efforts to develop a minimally invasive, growth-preserving method are both timely and commendable. However, I believe that one critical issue – namely the long-term safety of transphyseal drilling in adolescents – requires further discussion and investigation before this technique can be widely accepted as definitively “safe.” I have been assured that the article complies with TITAN Guidelines 2025-governing declaration and use of AI[2].

Firstly, while the authors report no complications at an average follow-up of 29.8 months, I contend that this timeframe may be insufficient to detect late-onset physeal disturbances. The proximal tibial physis often remains active beyond 16–18 years of age, particularly in female or delayed-growth patients. Growth disturbances such as angular deformities, limb-length discrepancies (LLD), or tibial recurvatum may manifest only after skeletal maturity is fully achieved, which frequently occurs beyond the study’s surveillance window.

Supporting this concern, a 2023 systematic review analyzing over 2000 pediatric anterior cruciate ligament (ACL) reconstructions reported a pooled 2.6% incidence of growth disturbances – some of which emerged years after surgery, even when tunnel diameters were ≤6 mm[3]. Furthermore, Johnson *et al* recently emphasized the need for long-term monitoring in patients undergoing limited transphyseal ACL techniques, noting that even small tunnels (4–5 mm) carry a measurable risk of physeal injury[4]. This view is supported by Ziegler *et al*, who reported that although growth-plate-sparing ACL reconstruction reduces the risk, a residual possibility of growth disturbance remains and should not be ignored[5]. The 3.4 mm transphyseal tunnel used in the current study, although relatively small, still traverses the physis completely and removes a nontrivial proportion of physeal cross-sectional area. Therefore, to conclude that this technique “minimizes interference with growth plates”[1] may be premature without extended, high-quality follow-up.

Secondly, I believe that the absence of long-term physeal outcome data directly challenges the authors’ core conclusion. If growth abnormalities or asymmetries were to appear several years postoperatively, the foundational claim of procedural safety would be undermined. In this regard, the study’s limitations are not merely academic – they may materially impact the technique’s risk-benefit calculus, particularly when counseling families about surgical options.

Thirdly, I suggest several strategies to close this knowledge gap. A prospective, multicenter registry with serial clinical and radiographic assessments – ideally utilizing low-dose EOS imaging or full-length standing radiographs – would allow for objective detection of angular changes or LLD as small as 3°. Additionally, emerging tools such as MRI-based physeal mapping and high-resolution ultrasound may help detect early physeal bar formation before clinical deformities arise. Finally, future comparative studies including physeal-sparing anchor-based techniques could elucidate whether the theoretical benefits of suspensory fixation truly outweigh any latent risks to growth.

Fourth, while I commend the authors for assessing postoperative knee stability, I note that stability was determined solely by manual examination. The inclusion of instrumented assessments, such as the Rolimeter, KT-1000, or stress radiography, would have strengthened the evidence that the elastic fixation method provides durable mechanical integrity – especially during the dynamic adolescent growth phase. As posterior instability often presents subtly, objective quantification would enhance both internal and external validity.

From a clinical perspective, the proposed technique offers several advantages: arthroscopic precision, minimal invasiveness, and no need for direct hardware fixation into the fracture bed. Nevertheless, surgeons should be cautious in interpreting short-term outcomes as definitive evidence of long-term safety. Until more robust longitudinal data are available, I recommend a shared decision-making model that transparently presents both the benefits and the unresolved uncertainties associated with transphyseal suspensory fixation in growing patients.

In conclusion, the arthroscopic technique described by Chen *et al* represents an innovative and potentially valuable treatment for adolescent PCL avulsion fractures. However, the long-term consequences of physeal transgression have not yet been fully addressed. I urge the authors – and the orthopedic community at large – to pursue extended, objective follow-up studies to ensure that the early functional success of this technique does not come at the cost of delayed growth complications. While some prior studies have reported an absence of growth disturbances following PCL surgery in pediatric populations[6], such results are typically based on open techniques or short-term outcomes and should not be extrapolated uncritically to transphyseal arthroscopic approaches. Only by resolving this key issue can the procedure’s long-term safety and efficacy be affirmed.

## Data Availability

The datasets used and/or analyzed during the current study are available from the corresponding author on reasonable request.

## Research registration unique identifying number (UIN)

This study did not require registration in a publicly accessible database, as it did not involve human participants prospectively enrolled in a trial.
